# Imaging of the medial rectus muscle predicts the development of optic neuropathy in thyroid eye disease

**DOI:** 10.1038/s41598-022-10043-z

**Published:** 2022-04-15

**Authors:** Marcel Berger, Juliane Matlach, Susanne Pitz, Manfred Berres, Franz Axmacher, George J. Kahaly, Marc A. Brockmann, Matthias Müller-Eschner

**Affiliations:** 1 Department of Neuroradiology, University Medical Center of the Johannes Gutenberg-University of Mainz, Mainz, Germany; 2 Department of Ophthalmology, University Medical Center of the Johannes Gutenberg-University of Mainz, Mainz, Germany; 3 Orbital Center, Bürgerhospital, Frankfurt, Germany; 4 Department of Mathematics and Technology, University of Applied Sciences Koblenz, Remagen, Germany; 5 Department of Medicine I, University Medical Center of the Johannes Gutenberg-University of Mainz, Mainz, Germany

**Keywords:** Autoimmune diseases, Eye diseases, Diagnostic markers, Eye manifestations

## Abstract

Goal of the study was to evaluate bony orbit remodeling and extraocular muscle (EOM) volume in thyroid eye disease (TED) and their role as predicting factors for development of dysthyroid optic neuropathy (DON). Orbital computed tomography of 92 patients with TED with (76 orbits) or without DON (98 orbits) were retrospectively evaluated. Orbits (n = 40) of subjects without TED served as controls. Measurements of the bony orbit as well as EOM volume were incorporated into a generalized linear mixed model to predict DON. The angle of the medial orbital wall was significantly smaller (*p* < 0.001) in patients with TED (− 2.3 ± 3.6°) compared to patients with TED + DON (1.0 ± 4.1°). Both groups differed significantly from controls (− 4.2 ± 2.7°). Bowing of the medial orbital wall correlated positively with muscle volume (*r* = 0.564; *p* < 0.001). Total EOM volume was significantly larger in TED + DON (7.6 ± 2.5cm^3^) compared to TED only (5.6 ± 3.0cm^3^; *p* < 0.001) or controls (2.6 ± 0.5cm^3^). Multivariate analysis revealed the medial rectus muscle volume (TED: 1.06 ± 0.48cm^3^ vs. TED + DON: 2.16 ± 0.84cm^3^) as the strongest predictor, achieving a specifity of 86.7% and a sensitivity of 73.7% in diagnosing DON in univariate analysis. Though characterized by a wide range of variability, increased medial rectus muscle volume is the strongest predictor for DON in our patient cohort with TED when analyzing a single muscle.

## Introduction

Thyroid eye disease (TED) is the most common extrathyroidal manifestation of autoimmune hyperthyroidism, occurring in 30–50% of cases e^1,2^. TED is not limited to patients affected by Graves’ disease, but may also be observed in Hashimoto's thyroiditis, and rarely also without thyroid autoimmunity (euthyroid orbitopathy)^3–6^.

In TED, autoimmune-induced stimulation of orbital fibroblasts leads to increased production of hydrophilic mucopolysaccharides i.e., hyaluronic acid, secretion of pro-inflammatory cytokines, and enhanced adipogenesis hence causing edema and expansion of orbital connective tissue^7,8^.

The aforementioned changes increased volume and pressure within the bony orbital cavity are responsible for most mechanical problems in TED^9^. Inflammation, edema and swelling of the extraocular muscles impair ocular motility with subsequent diplopia.

A serious complication of TED is the occurrence of dysthyroid optic neuropathy (DON) in approximately 5% of cases. DON is characterized by reduced visual function/acuity, color perception and visual field defects which may progress to permanent loss of vision if untreated^10,11^. The most widely accepted cause of DON is the compression of the optic nerve by expansion of the enlarged extraocular muscles and—to a lesser extent—orbital fat, causing ischemia and inhibition of axonal flow^7^.

Earlier studies analyzed orbital bone dimension and size of extraocular muscles using computed tomography (CT). However, uncertainty remained regarding the association of bowing of the medial wall in DON, as some studies reported such an association^12,13^, while others did not^14,15^.

As volumetric analyses seem superior^15,16^ over plain measurement of muscle diameter to predict DON, we decided to combine volumetric measurements with angular measurements of the orbit as previously described al.^13,14^, in order to improve risk stratification for patients with TED to develop DON.

## Material and methods

### Study population

**Figure 1:**
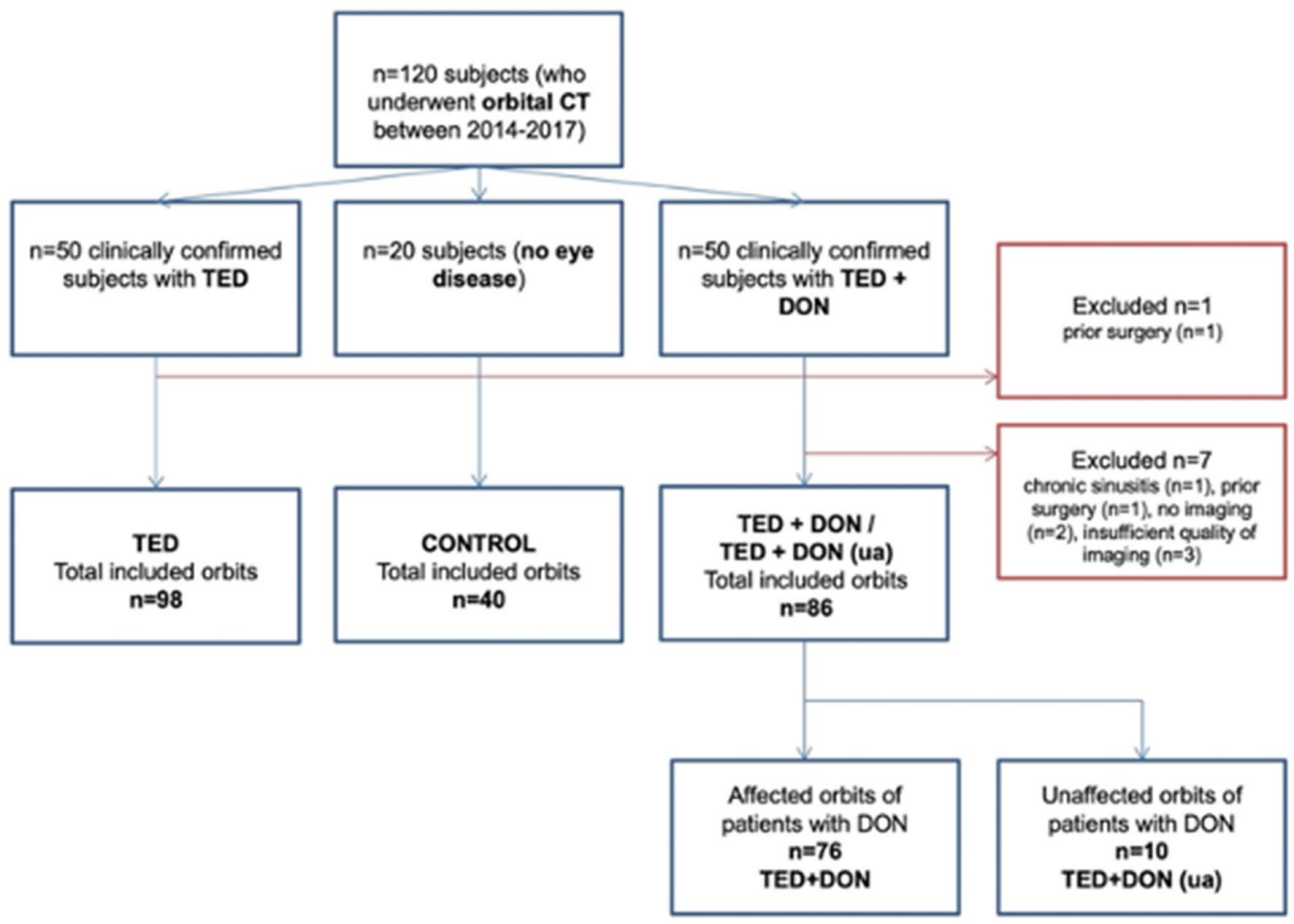
Flowchart of patient inclusion and exclusion in this study.

Orbital CT scans of 92 patients with clinically diagnosed TED (184 orbits) acquired 2014–2017 were retrospectively analyzed (Fig. 1). All patients were clinically diagnosed with TED according to the defined clinical criteria of the European Group on Graves’ Orbitopathy (EUGOGO)^2^.

Of these 92 patients with clinically diagnosed TED, 49 patients (98 orbits, 26 male 72 female, mean age 49 ± 13.8 years) were allocated to the TED only group (without DON). DON was diagnosed in 43/92 patients (86 orbits). Of these, 76 orbits (59 female, 17 male, mean age 60 ± 12.5 years) were allocated to the TED + DON group. Optic nerve dysfunction was considered present if at least two of the following tests were impaired: visual acuity, pupillary light response, color vision/saturation and perimetry; patients with other causes for the impairment of visual function were not included in this population^10^. Patients with other causes for the impairment of visual function were not included in this population^10^. Visual acuity in logMAR was 0.51 ± 0.51 (0.00–3.00) in patients with DON, with two patients being able to only count fingers or to see hand movement. 30 eyes of 76 patients with DON (39.5%) had a relative afferent pupillary defect. Mean defect was 10.86 ± 7.22 dB using automated static perimetry.

In 10 patients DON was unilateral. This unaffected (ua) eye was measured as well and formed its own group (TED + DON (ua)) (10/86 orbits, 3 male, 7 female, mean age 61 ± 12.6 years).

None of the assessed patients had any prior ophthalmic or sinusal surgery.

We furthermore analyzed the orbits of 20 subjects (40 orbits, 28 female, 12 male, mean age 57 ± 8.9 years), who served as control. These CT scans were performed to e.g. rule out intracranial hemorrhage or ischemia. None of these subjects had skull or face injuries, a history of thyroid or orbital disease or any other condition that could interfere with proper measuring.

The study protocol was approved and consent was waived by the local ethics committee (Landesärztekammer Rhineland-Palatine, #2021-15977). The study was conducted according to state laws. All research was performed in accordance with relevant local guidelines and/or regulations and in accordance with the declaration of Helsinki.

### Radiologic analyses

All orbital CT scans were part of a routine clinical protocol performed on a Toshiba Aquilion 32 slice CT (Toshiba Medical Systems Group Co, Zoetermeer, The Netherlands). The acquisition parameters used were as follows: 120 kV, 200 mA, 210 mm display FOV, pitch of 21 and a slice thickness of 0.5 mm.

**Figure 2:**
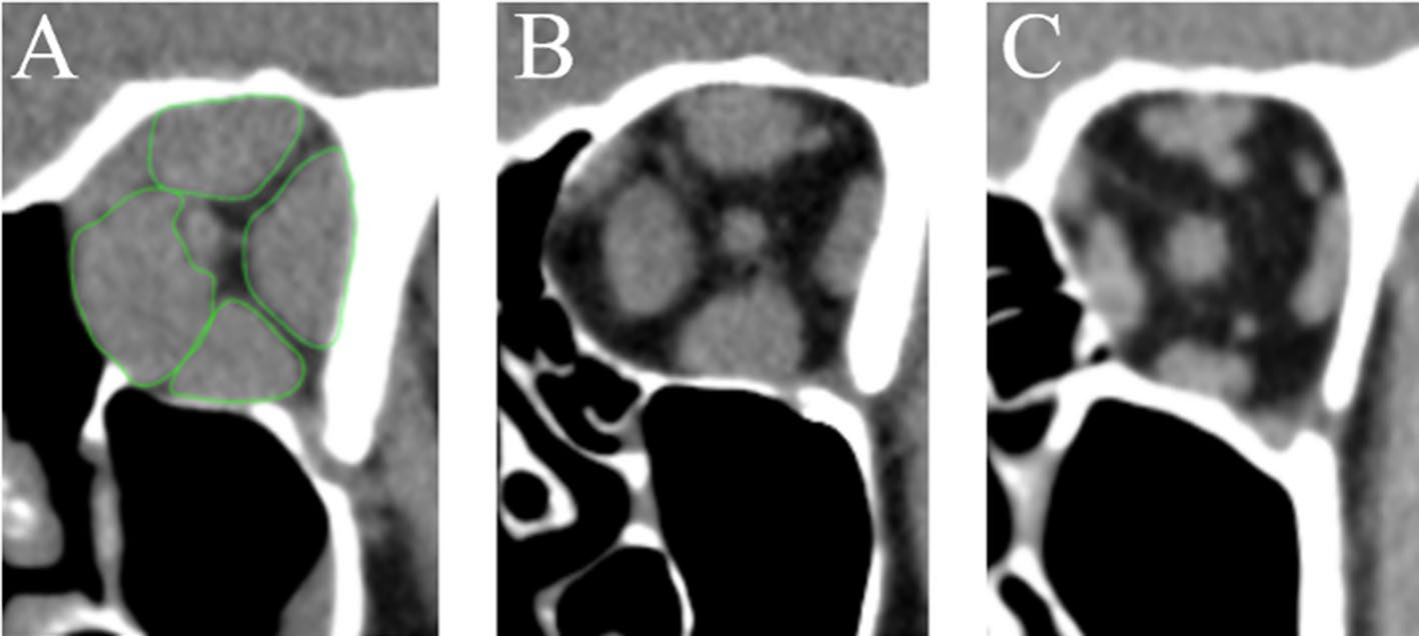
Coronal reconstructed CT images (orthogonal to the optic nerve) (**A**) patient with TED + DON and massive enlargement of the EOM (measured muscles exemplary outlined in green); (**B**) patient with TED only and substantial increase in muscle volume; (**C**) healthy control group orbit with normal muscle dimensions.

**Figure 3:**
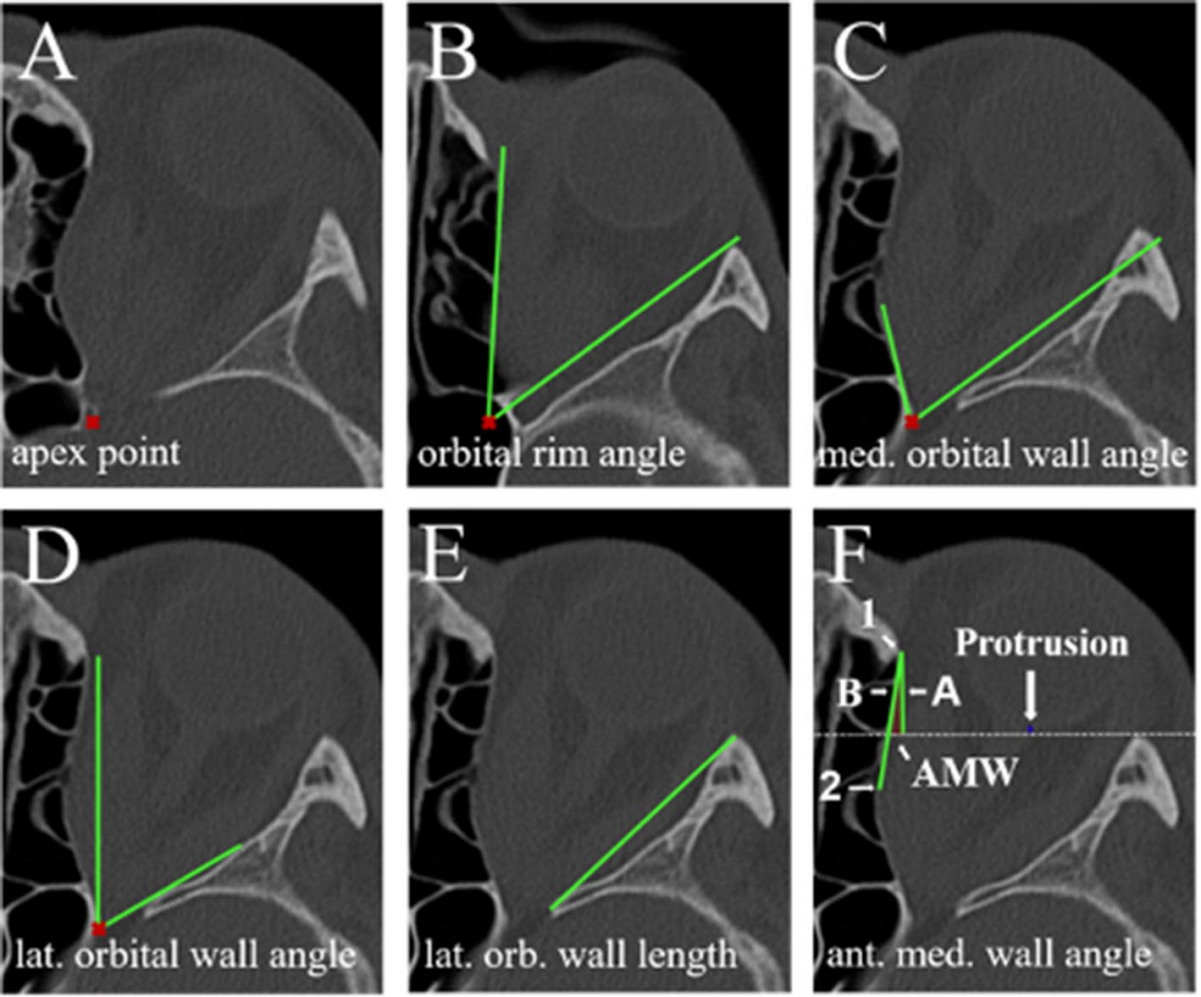
Angle and protrusion measurements: (**A**) apex point, (**B**) orbital rim angle, (**C**) lateral orbital wall angle, (**D**) medial orbital wall angle, (**E**) length of the lateral orbital wall, (**F**) anterior medial wall angle, (**G**) AMW = anterior medial wall angle (see chapter “Angular measurements” for further details of parameter assesment).

To ensure consistency throughout all measurements, the measurements were taken on reconstructed 2 mm slices to be either orthogonal to the optic nerve (muscle volume measurements) (Fig. 2) or parallel to the infraorbitomeatal line (bony orbit measurements) (Fig. 3).

### Angular measurements

Orbital angles and length of the lateral orbital wall, as well as medial wall measurements were performed as described in recent studies^13,14^.

The apex point was defined as the anterolateral border of the groove in the sphenoid body formed by the intracavernous portion of the internal carotid artery (red cross in Fig. 3A). From this point, the orbital rim angle was measured on the height of the medial palpebral ligament (angle between green lines in Fig. 3B). The medial and lateral orbital wall angles were assessed on the image where the medial and lateral rectus muscle had the widest diameter as angular change between the orbital rim angle and the widest point of the orbital wall (Fig. 3C,D). The length of the lateral orbital wall was measured just below the anterior clinoid process (Fig. 3E).

To assess the anterior medial wall angle, a perpendicular line was drawn from the beginning of the medial orbit wall on the nasal bone (Fig. 3F, point 1) perpendicular to the interzygomatic line. This was considered line A (Fig. 3F, A). Line B (Fig. 3F, B) was drawn, connecting point 1 with the point of maximum excursion of the medial wall (Fig. 3F, point 2). The resulting angle formed between line A and line B was considered the anterior medial wall angle (Fig. 3F, AMW). This angle was considered positive if the point 2 was lateral to line A or negative, if medial to it. Exophthalmos/Protrusion was measured as the distance between the interzygomatic line and posterior sclera just lateral of the optic nerve head (blue lines, Fig. 3F).

### Volumetric analyses of the extraocular muscles

Volume of the extraocular muscles was measured using the closed polygon tool of OsiriX version 9.0 (Aycan Digitalsysteme GmbH, Würzburg, Germany) by outlining the profile of the muscles on coronal images. Due to difficulties to accurately discern the levator palpebrae from the superior rectus muscle, these muscles were evaluated as the superior muscle group.

### Statistical tests

Multiple statistics were conducted with IBM SPSS Statistics for Windows, version 25 (IBM Corp., Armonk, N.Y., USA) using linear mixed models to compare muscle volumes and angles between patient groups and a generalized linear mixed model to predict DON from these measurements. All available variables that were distinctly different (*p* < 0.05) on univariate analysis were then encompassed in a multivariate model. No adjustment for multiple testing was done.

## Results

**Figure 4:**
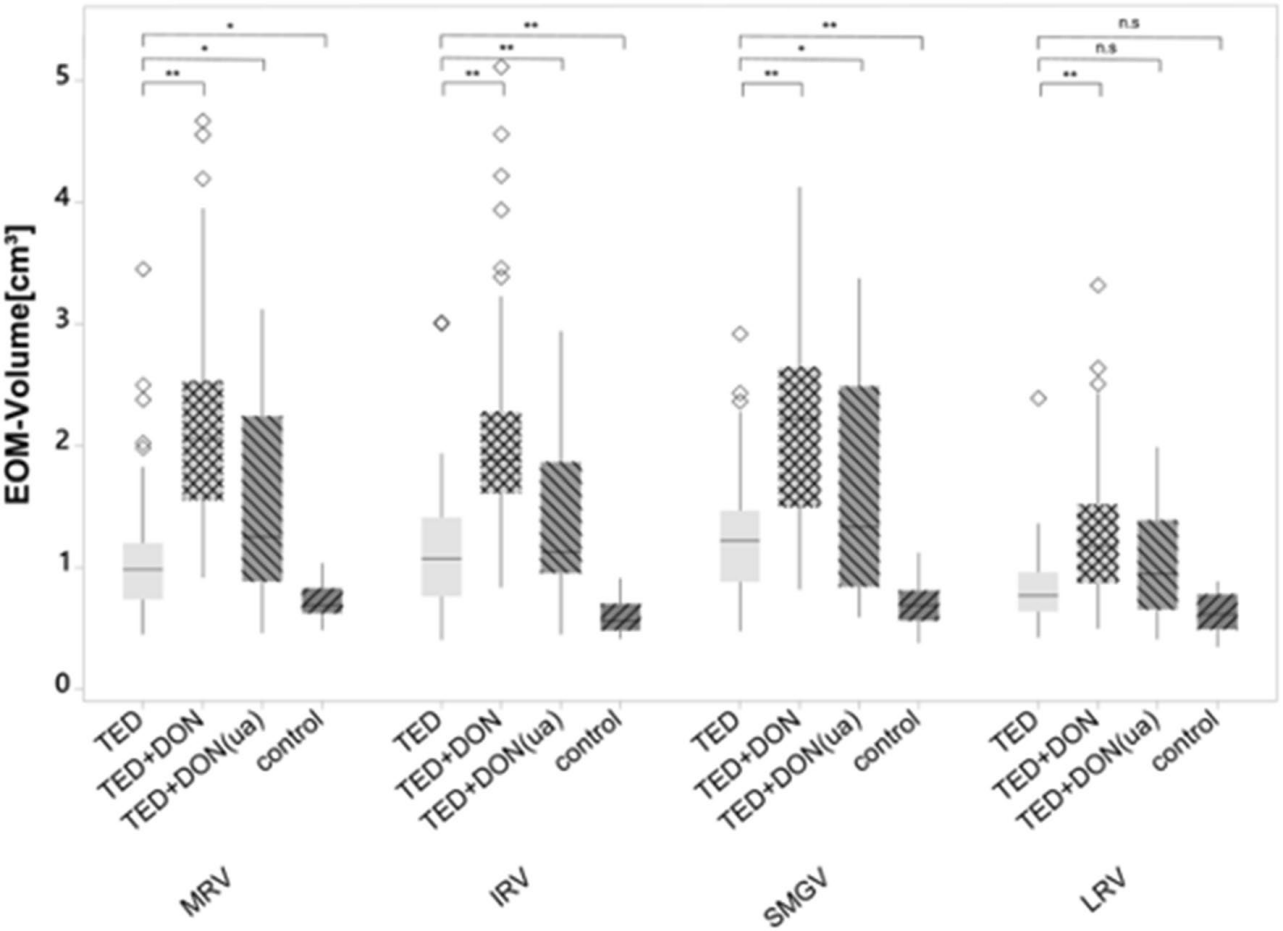
Boxplots showing the differences between muscle volumes of each group. MRV (medial rectus muscle volume) IRV (inferior rectus muscle volume) SMGV (superior muscle group volume) LRV (lateral rectus muscle volume). *Indicates p < .05, **indicates p < .001.

Disease duration was significantly shorter and clinical activity score was significantly higher in patients with TED + DON compared to patients with TED only (disease duration: 2.1 ± 3.1 vs. 4.6 ± 5.2 years, p = 0.006; CAS: 1.9 ± 1.1 vs. 3.9 ± 1.2, p < 0.001). Likewise, ocular motility was restricted significantly stronger in patients with TED-DON compared to TED only (Table 1). Compared to patients with TED only, the medial, inferior, lateral, and the superior muscle group volume were significantly higher in the TED + DON group using the univariate mixed model analysis (all *p* < 0.001). Muscle volume of the TED group was significantly larger (*p* < 0.005) compared to the control group in all measured muscles, except for the lateral rectus muscle (*p* = 0.055, Table 1, Fig. 4).

**Table 1:** Muscle and angle measurements of the different groups and ophthalmological patients’ characteristics.

TED	TED + DON (*N* = 76)	TED *(N* = 98)	*p*	TED + DON(ua) (*N* = 10)	Control *(N* = 40)
**Volume [cm**^3^**]**					
Medial rectus muscle	2.16 ± 0.84	1.06 ± 0.48	< 0.001	1.56 ± 0.91	0.73 ± 0.15
Inferior rectus muscle	2.04 ± 0.81	1.10 ± 0.49	< 0.001	1.39 ± 0.75	0.59 ± 0.13
Superior muscle group	2.16 ± 0.47	1.23 ± 0.47	< 0.001	1.62 ± 0.97	0.67 ± 0.23
Lateral rectus muscle	1.28 ± 0.55	0.81 ± 0.26	< 0.001	1.03 ± 0.49	0.63 ± 0.16
Total muscle volume	7.64 ± 2.52	4.20 ± 1.52	< 0.001	5.60 ± 3.01	2.62 ± 0.49
**Bony orbit measurements [degrees)**					
Orbital rim angle	45.67 ± 2.81	45.58 ± 2.80	0.919	45.37 ± 3.90	44.19 ± 2.75
Medial angle change	0.98 ± 4.09	− 2.30 ± 3.64	< 0.001	− 1.95 ± 4.94	− 4.22 ± 2.71
Medial wall angle	46.66 ± 5.47	43.28 ± 4.72	< 0.001	43.42 ± 6.66	39.97 ± 3.4
Lateral angle change	4.9 ± 2.72	4.68 ± 2.49	0.598	6.08 ± 2.78	4.23 ± 2.30
Lateral wall angle	50.57 ± 3.90	50.25 ± 4.12	0.680	51.45 ± 4.66	48.42 ± 3.33
Lateral wall length [cm]	4.25 ± 0.27	4.18 ± 0.29	0.236	4.12 ± 0.30	4.25 ± 0.31
Proptosis [cm]	0.05 ± 0.30	− 0.16 ± 0.32	< 0.001	− 0.15 ± 0.43	− 0.59 ± 0.29
Anterior medial angle	2.75 ± 4.52	5.99 ± 4.70	< 0.001	4.98 ± 4.54	6.82 ± 3.22
**Diagnosis**					
Graves’ disease	41 (95.3%)	48 (98%)	0.205	9 (90%)	
Hashimoto’s thyreoiditis	2 (4.7%)	0		1 (10%)	
Other	0	1 (2%)		0	
**Disease severity**					
Mild	0 (0%)	31 (33.0%)	< 0.001	5 (50.0%)	
Moderate-to-severe	0 (0%)	65 (67.0%)		5 (50.0%)	
Sight-threatening (DON)	76 (100%)	0 (0%)		0 (0%)	
Disease duration (years)	2.1 ± 3.1	4.6 ± 5.2	0.006	2.9 ± 3.3	
Smokers	16 (37.2%)	16 (32.7%)	0.667	2 (20.0%)	
**Clinical activity**					
Inactive	8 (10.8%)	72 (76.6%)	< 0.001	5 (50.0%)	
Active	66 (89.2%)	22 (23.4%)		5 (50.0%)	
Clinical activity score (CAS)	3.9 ± 1.2 (2–7)	1.9 ± 1.1 (0–5)	< 0.001	2.3 ± 1.9 (0–6)	
Proptosis (Hertel) [mm]	22.2 ± 4.0 (12–33)	22.0 ± 3.2 (14.5–31)	0.610	19.9 ± 4.2 (12–27)	
**Diplopia**					
None	13 (36.1%)	13 (28.3%)	0.835	4 (44.4%)	
Intermittent	3 (8.3%)	5 (10.9%)		1 (11.11%)	
Gaze-dependent	10 (27.8%)	12 (26.1%)		4 (44.4%)	
Constant	1027.8%)	16 (34.8%)		0 (0%)	
**Ocular motility**					
Abduction	27.5 ± 12.1 (− 5–40)	39.2 ± 10.1 (10–50)	< 0.001	3.1 ± 11.3 (10–50)	
Elevation	13.7 ± 12.0 (− 5–40)	27.0 ± 13.9 (− 20–45)	< 0.001	22.2 ± 12.9 (0–40)	
Upper lid retraction [mm]	1.1 ± 1.3 (0–6)	0.9 ± 1.4 (0–5)	0.486	0.7 ± 1.1 (0–3)	
Lower lid retraction [mm]	0.5 ± 0.9 (0–5)	0.7 ± 1.1 (0–4)	0.345	0.6 ± 1.0 (0–3)	
Lid lag [mm]	0.8 ± 1.1 (0–4)	0.2 ± 0.7 (0–3.5)	< 0.001	0.1 ± 0.3 (0–1)	
Lid aperture [mm]	11.5 ± 2.9 (5–19)	11.9 ± 2.4 (7–18)	0.400	10.1 ± 2.7 (5–15)	

p-values between TED + DON and TED group (t-test or Fisher exact test).

All descriptives values are mean ± SD or absolute values (%).

The muscle volume of each muscle of the unaffected orbits of DON patients (TED + DON (ua) group) was markedly decreased compared to those affected by DON (TED + DON group; e.g. medial rectus volume (MRV) *M* = 1.56 cm^3^ vs 2.16 cm^3^). However, compared with the TED group or the control group, the TED + DON (ua) group showed a distinctly increased muscle volume for all muscles (e.g. MRV: M = 1.56 cm^3^ vs. M = 1.06 cm^3^; p = 0.015; vs. M = 0.73 cm^3^; p < 0.001) apart from that of the lateral rectus muscle (M = 1.03 cm^3^ vs M = 0.81 cm^3^; p = 0.603 vs M = 0.63 cm^3^; p = 0.062).

With regard to the bony boundaries of the orbit, differences in the medial orbital wall angle were noticeable. Patients with TED + DON showed a more concave medial wall than patients of the TED group (*M* =  + 0.98° vs. − 2.3°; *p* < 0.001) or the TED + DON (ua) group (*M* = − 1.95°*, p* < 0.001). The medial wall angle also differed significantly between the control group and patients of the TED group (*M* = − 4.22° vs. − 2.3°; *p* = 0.03).

The lateral wall angle, orbital rim angle, as well as the length of the lateral orbit wall did not differ markedly between the TED + DON, TED or TED + DON (ua) groups. Neither was there a difference when comparing the control group with the TED + DON group and the TED group.

Both types of angle measurement of the medial orbital wall showed the correlation between increased muscle volume and bowing of the medial wall. The medial rectus muscle had a slightly higher correlation (*r* = *0*.564) compared to the other muscles or to the total muscle volume (Table 2).

**Table 2:** Pearson correlation between the curvature of the medial orbital wall as well as the extent of proptosis and muscle volume.

	Medial angle change (MAC)	Anterior medial wall angle (AMW)	Distance between interzygomatic line and posterior sclera
Medial rectus volume	0.564*	− 0.473*	0.595*
Inferior rectus muscle	0.474*	− 0.355*	0.662*
Superior muscle group	0.518*	− 0.376*	0.635*
Lateral rectus muscle	0.411*	− 0.335*	0.597*
Total muscle volume	0.542*	− 0.423*	0.674*

*Indicates p < .001.

In the generalized linear mixed model, which contained all muscle measurements with distinct differences (i.e. *p* < 0.05) between the TED + DON and TED groups, the medial rectus muscle was the most influential variable regarding diagnostic accuracy (specificity: 86.7%, sensitivity: 73.7%). Adding all other muscles resulted in a specificity of 87.8% and a sensitivity of 77.6%. Further addition of the medial wall angle did not confer any diagnostic benefit.

**Figure 5:**
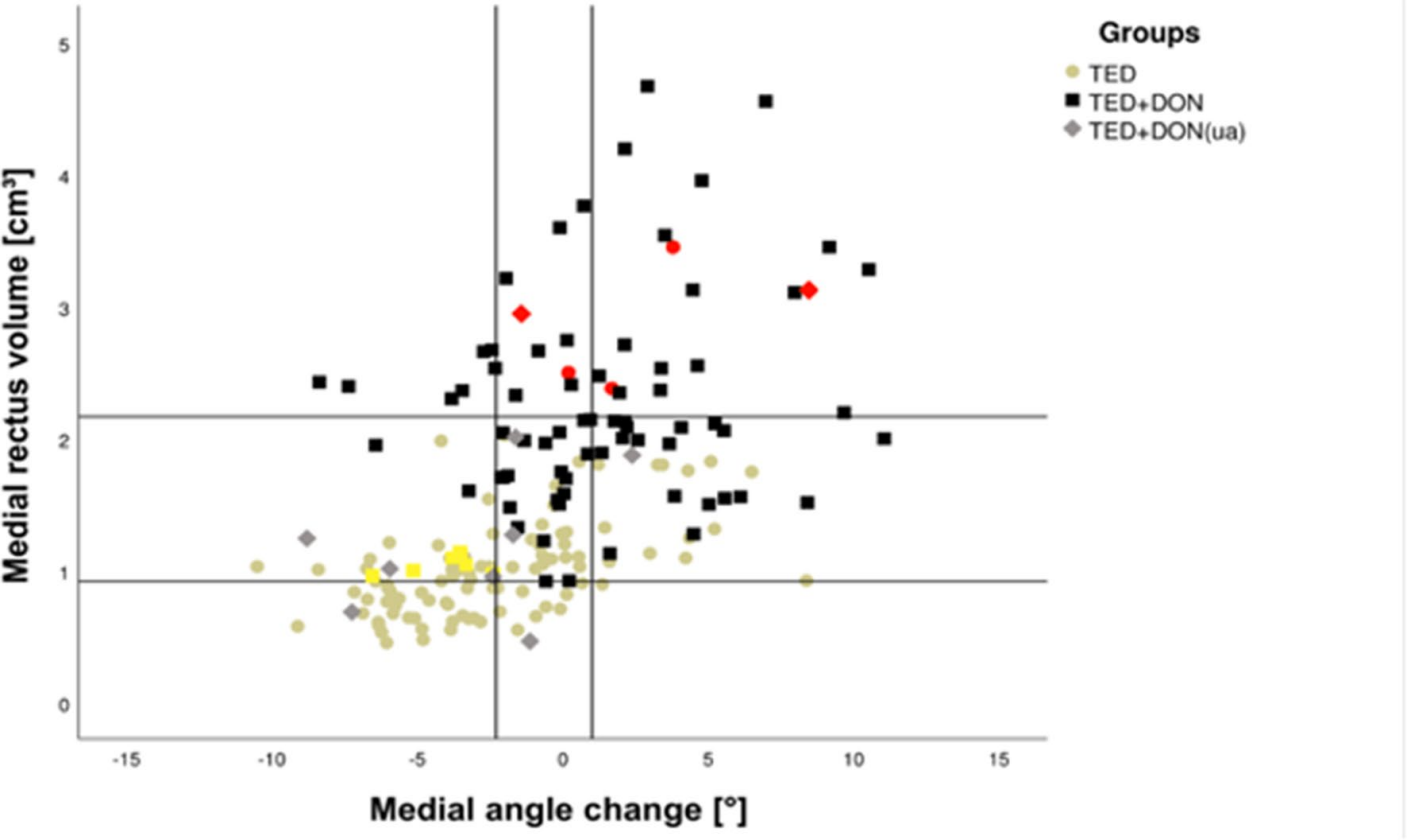
Scatterplot showing medial rectus volume (MRV) as well as the medial angle change (MAC). Upper horizontal line shows the average MRV of the TED + DON group (2.16 cm^3^). Lower horizontal line is set at the lowest MRV at which DON occurred (0.92 cm^3^). Right vertical line is showing the average MAC of DON patients, the left vertical line shows the average MAC of the TED group. Patients with higher than average MRV but not affected by DON are marked red. Yellow squares are marking DON-patients with a similar MRV and smaller MAC than the average of the TED group.

A few subjects (*n* = 5, Fig. 5, marked red) stood out in that they had a higher MRV than that of the TED + DON group without, however, being affected by DON. There are also patients with DON (n = 6, Fig. 5, marked yellow) having a similar MRV and a smaller medial wall angle compared to patients with TED only.

## Discussion

Radiological characteristics in patients with DON have been a major subject of research. Changes such as enlargement of the superior ophthalmic vein, anterior displacement of the lacrimal gland or the presence of an intracranial fat prolapse have been investigated^17–19^. The most thoroughly investigated radiological parameter aiding the diagnosis of DON is the increase of extraocular muscle size, ultimately leading to apical crowding and DON.

In the underlying study we have been able to confirm an increase of muscle volume especially that of the medial rectus muscle, to be the strongest predictor for the development of DON in patients with TED. Although dimensions of the bony orbit significantly differed between the examined groups, there was no difference predisposing to the development of DON in patients with TED prior to disease-related bony orbital changes.

In other studies apical crowding was graded on a categorical scale or by forming the ratio of the diameters of the horizontal or vertical extraocular muscles and the diameter of the bony orbit^20,21^. Other authors calculated the ratio between soft- and fat tissue around the apex area to form a volumetric apical crowding index^16^.

To our knowledge, the underlying study is the only one including both, measurements of individual muscle volumes as well as of the bony orbit. Furthermore, our collective of DON patients seems to be the largest hitherto analyzed. Finally, previously published research ignored the unaffected orbits of patients with DON, which in our study were analyzed as a separate subgroup, as it cannot be excluded that relevant anatomical differences prior to disease manifestation in orbits of patients with DON and those without exist.

With the medial orbital wall being much thinner than the lateral orbital wall and the air-filled ethmoidal sinuses providing less resistance, the medial wall is more prone to bony remodeling. This thesis is supported by the fact that the change of the medial orbital angle correlates with an increase in muscle volume, especially that of the medial rectus muscle as shown in the TED + DON-group. Our findings regarding muscle volume are consistent with previously published research^13,15^, showing the volume or thickness of the medial rectus muscle to be the most significant predictor for the development of DON, which is most likely due to its close anatomic relation to the optic nerve in the optic canal^15^. At some point during the process of muscle enlargement, compensatory mechanisms of the orbit (mainly the expansion into the ethmoidal sinuses) seemed to exceed their limits leading to a critical increase of pressure within the orbit and ultimately optic nerve damage.

Subjects in the TED + DON group showed a distinctly increased medial orbital angle compared to the TED group. The angles of both groups were also significantly increased compared to the control group. This is in accordance with other studies^12,13^. Adding medial orbital angle change to our multivariate analysis, however, did not improve the diagnostic accuracy. This suggests that the increased bowing of the medial wall serves as a surrogate parameter for the increase in muscle volume. Interestingly, this finding is contrary to previous studies^14,15^, in which there was no significant difference in medial orbital wall curvature between these groups. Reasons for this might be the use of subjective evaluation methods to assess the bowing of the medial orbital wall^15^, the higher number of subjects in our DON group, or the extent of muscle enlargement inherited by them.

Previous findings describing an increase of the lateral wall angle in patients suffering from DON could not be replicated in our study. While subjects allocated to the TED + DON group had a lateral wall angle larger than subjects of the TED group, the difference was not significant (*p* = 0.6). Reasons for this could be the larger sample size of our study, the different patient ethnicity, group composition or even more increased bowing of the medial wall resulting in a reduction of pressure on the lateral wall. The fact that lateral orbital wall remodeling occurs later during disease progression could also explain this finding.

Unaffected (ua) orbits of DON patients did not differ markedly from orbits of the TED group regarding the dimension of the bony orbit. Despite the small sample size and the assumption, that both orbits developed more or less equally, this data might suggest that there are no characteristic bony differences or that these are too minuscule to measure, which would affect the development of DON patients prior to thickening of the muscles.

Besides bony orbital changes, protrusion of the eyeball is another effect of increased intra-orbital volume. Proptosis was remarkably higher in DON patients and correlated well with muscle volume (Table 1). The scatterplot (Fig. 5) of medial rectus volume (MRV) and medial angle change (MAC) of DON and TED + DON patients demonstrates, that a sharp delineation between these two groups is not possible and that some cases (red and yellow points) develop atypically. Thus, these two groups therefore cannot be easily distinguished from the non-affected, respectively affected population on the basis of the measurements obtained and illustrate the difficulty of diagnosing DON using anatomical radiological data. Thus, alternative approaches like radiomics or diffusion tensor imaging might offer interesting approaches and are currently being investigated. However, currently, the clinical ophthalmological examination is indispensable and increases sensitivity and specificity^22^.

Our data confirms the heterogeneity of the disease with several existing subtypes^23^. While most of the subjects exhibiting DON are showing a distinct increase in muscle volume, a subset shows no or barely any increase. On the other hand, our data shows a trend towards discrimination of both groups (TED and TED + DON) and in our opinion it may be advisable, that patients with known TED and a medial rectus volume higher than 0.9cm^3^ should be monitored more closely disregarding the dimensions of the bony orbit, as this was the lowest value in our population associated with the development of DON (Fig. 5, lower horizontal line). The heterogeneity of TED has also been emphasized by Uddin et al., who stressed TED to be heterogeneous in its underlying pathogenesis, clinical manifestations, and response to medical and surgical treatment modalities^24^. In their manuscript they criticized that several previous categorizations of the clinical appearance of TED were dichotomous and underrepresented the heterogeneity of the disease. Consequently, Uddin et al. suggested a new classification system dividing TED into six different classes based on the phenotypic features (clinical and radiologic) and their response to different treatments.

Heterogeneity has not only been reported for clinical and radiological findings in EO, but also different imaging modalities including MRI and PET (alone or in combination with MRI or CT) have been used to study various morphological and functional parameters. In a recently published work, Weber et al. reported PET-MRI to be suited for the assessment of EO inflammation and to be a good discriminator for severe vs. mild-to-moderate EO^25^. A variety of other parameters have been analyzed in EO in numerous other studies. These were mainly morphological parameters like the volume or width of fat or muscles^26^ or the optic nerve^27^, herniation of the lacrimal gland^28^, contrast agent uptake and T2-hyperintensity (as surrogate parameters for inflammation)^29,30^, quantitative MRI and T1-mapping of eye muscles^31,32^, just to name a few. Therefore imaging has a significant role in the investigation of TED. Siakallis et al., however, correctly pointed out that a consensus on the use of the different imaging modalities in the course of disease has not yet been reached^33^.

As it is uncommon to routinely perform CT or MRI of the orbit prior to the occurrence of TED, a definitive conclusion about risk factors regarding the dimensions of muscles and the bony orbit aggravating the progression from mere TED to TED + DON cannot be made based on this retrospective study. Thus, a follow up study closely assessing changes in muscle volume in our set of TED patients might be of interest to identify novel parameters (such as an increase in medial rectus volume in a certain amount of time) which are better suited to stratify the risk to progress from TED to TED + DON.

It should be noted that the gender ratio in our study differs from other publications. In our population the proportion of males in the DON group was noticeably lower than in comparable studies^13,16,20^. It might be noteworthy that inclusion of patients with mild and inactive disease in the TED group (compared to the moderate to severe and sight-threatening cases) can be a confounding factor. As our center primarily sees patients who have a more serious course of disease and for whom a CT scan is justified, the measured values might not necessarily be comparable to those of the entire TED population. DON, in the majority of cases, is a clinical diagnosis being based on abnormalities in assessment of visual acuity, color vision/color saturation, perimetry, fundoscopy and/or by testing of the pupillary light reaction/relative afferent pupillary defect in patients with TED. Radiological imaging while not mandatory, should aid in making a diagnosis in borderline cases or help to identify patients with an increased risk for the development of a DON.

To conclude, the underlying study shows the complexity of DON and the inherent problem of identifying patients with TED to develop DON. From our experience volumetric measurements of the medial rectus muscle seem to provide the best sensitivity and specificity for the development of DON and patients with a volume of the medial rectus muscle of more than 0.9cm^3^ should be monitored closely.

## Data Availability

The datasets (CT images) generated during and/or analyzed during the current study are not publicly available due to data privacy reasons.
